# Chemical screen identifies a geroprotective role of quercetin in premature aging

**DOI:** 10.1007/s13238-018-0567-y

**Published:** 2018-08-01

**Authors:** Lingling Geng, Zunpeng Liu, Weiqi Zhang, Wei Li, Zeming Wu, Wei Wang, Ruotong Ren, Yao Su, Peichang Wang, Liang Sun, Zhenyu Ju, Piu Chan, Moshi Song, Jing Qu, Guang-Hui Liu

**Affiliations:** 1 Advanced Innovation Center for Human Brain Protection, National Clinical Research Center for Geriatric Disorders, Xuanwu Hospital of Capital Medical University, Beijing, 100053 China; 2 National Laboratory of Biomacromolecules, CAS Center for Excellence in Biomacromolecules, Institute of Biophysics, Chinese Academy of Sciences, Beijing, 100101 China; 3 State Key Laboratory of Stem Cell and Reproductive Biology, Institute of Zoology, Chinese Academy of Sciences, Beijing, 100101 China; 4 State Key Laboratory of Membrane Biology, Institute of Zoology, Chinese Academy of Sciences, Beijing, 100101 China; 5 University of Chinese Academy of Sciences, Beijing, 100049 China; 6 Institute of Stem cell and Regeneration, Chinese Academy of Sciences, Beijing, 100101 China; 7 Key Laboratory of Regenerative Medicine of Ministry of Education, Institute of Aging and Regenerative Medicine, Jinan University, Guangzhou, 510632 China; 8 The MOH Key Laboratory of Geriatrics, Beijing Hospital, National Center of Gerontology, Beijing, 100730 China

**Keywords:** Quercetin, Stem cell, Aging, Werner syndrome, Hutchinson-Gilford progeria syndrome

## Abstract

Aging increases the risk of various diseases. The main goal of aging research is to find therapies that attenuate aging and alleviate aging-related diseases. In this study, we screened a natural product library for geroprotective compounds using Werner syndrome (WS) human mesenchymal stem cells (hMSCs), a premature aging model that we recently established. Ten candidate compounds were identified and quercetin was investigated in detail due to its leading effects. Mechanistic studies revealed that quercetin alleviated senescence via the enhancement of cell proliferation and restoration of heterochromatin architecture in WS hMSCs. RNA-sequencing analysis revealed the transcriptional commonalities and differences in the geroprotective effects by quercetin and Vitamin C. Besides WS hMSCs, quercetin also attenuated cellular senescence in Hutchinson-Gilford progeria syndrome (HGPS) and physiological-aging hMSCs. Taken together, our study identifies quercetin as a geroprotective agent against accelerated and natural aging in hMSCs, providing a potential therapeutic intervention for treating age-associated disorders.

**Electronic supplementary material:**

The online version of this article (10.1007/s13238-018-0567-y) contains supplementary material, which is available to authorized users.

## Introduction

Aging is associated with progressive functional decline over time at cellular, tissue and organismal levels, leading to increased vulnerability to diseases that include cancer, neurodegenerative disorders, as well as cardiovascular and metabolic diseases (Benayoun et al., 2015; Campisi, 2013; Kudlow et al., 2007; López-Otín et al., 2013). Possible mechanisms for aging include free radicals, telomere shortening (Harley, 1991), programmed senescence (Smith and Pereirasmith, 1996), genomic instability (Lombard et al., 2005), and loss of heterochromatin architecture (Benayoun et al., 2015; Kubben et al., 2016; Ren et al., 2017a; Ren et al., 2017b; Villeponteau, 1997; Zhang et al., 2015). On the basis of numerous studies having unraveled possible mechanisms of aging (Uccelli et al., 2008), continuous efforts are put into the identification of many novel targets with minimal side effects (López-Otín et al., 2013). In particular, drug screening for geroprotective chemicals has attracted curiosity and excited imagination throughout the history of humankind.

Progeroid syndromes are a group of rare genetic disorders characterized by clinical features of premature aging. Werner syndrome (WS) and Hutchinson-Gilford progeria syndrome (HGPS) are two best characterized types of progeria. WS is known as adult progeria, mainly characterized by premature aging pathologies associated with the degeneration of mesodermal tissues, resulting in symptoms such as osteoporosis and atherosclerosis (Opresko et al., 2003; Ozgenc and Loeb, 2006). WS is caused by mutated *WRN* gene, resulting in the loss of WRN protein expression. WRN participates in a continuum of cellular processes, spanning from DNA replication, transcription, repair, recombination as well as heterochromatin maintenance at telomeric and centromeric regions, thus pointing WS pathogenesis to genomic and epigenomic instability (Kudlow et al., 2007; Lebel, 2001; Li et al., 2016b; Wu et al., 2018; Zhang et al., 2015). The other well-studied progeria, HGPS is a devastating incurable disease with an average age of death at 14.6 years (Hennekam Raoul, 2006; Kreienkamp et al., 2016; Ullrich and Gordon, 2015). It is caused by the accumulation of progerin, a truncated protein encoded by a GGC>GGT (G608G) single-base mutation in *LMNA* gene instead of the functional nuclear lamina protein Lamin A by wild-type *LMNA* gene. HGPS patient-derived cells usually exhibit nuclear morphological abnormalities, altered signaling pathways, genomic and epigenetic instability and premature senescence (Burtner and Kennedy, 2010; Kubben et al., 2016; Kudlow et al., 2007; López-Otín et al., 2013; Liu et al., 2011a; Liu et al., 2011b; Polosak et al., 2011; Ren et al., 2017b; Wu et al., 2018; Yang et al., 2014).

WS and HGPS are both relevant disease models for understanding the mechanisms of aging and for finding effective treatments for aging-associated disorders. We previously reported that Vitamin C (VC) rejuvenates WS hMSCs by inducing a global change in the transcriptome of aging-related genes (Li et al., 2016b). In addition, Vitamin D is reported to improve HGPS cellular phenotypes by reducing progerin production and stabilizing key factors for maintaining genome integrity (Kreienkamp et al., 2016). We recently established a human stem-cell-based platform with isogenic WS and HGPS hMSCs with the identical genetic background for the comparative mechanistic studies of aging as well as the identification of new drug discovery strategies for progeria syndromes and physiological aging (Kubben et al., 2016; Li et al., 2016b; Liu et al., 2011a; Liu et al., 2011b; Wu et al., 2018; Zhang et al., 2015).

In this study, we screened a natural product library for geroprotective drugs using WS hMSCs and identified ten candidate compounds including quercetin. Mechanistically, quercetin alleviated hMSC senescence by promoting the self-renewal and differentiation abilities as well as restoring the heterochromatin architecture of WS hMSCs. RNA-sequencing analysis revealed that quercetin rejuvenated WS hMSCs via the regulation of multiple cellular processes related to cell cycle, chromosome condensation and anti-oxidation, which were of mechanistic commonalities for VC-mediated geroprotective effects in WS hMSCs. In addition, quercetin rejuvenated HGPS as well as physiological-aging hMSCs. Taken together, these data indicate quercetin as a geroprotective agent against premature and physiological human aging.

## Results

### Natural product screening using Werner syndrome hMSCs

**Figure 1:**
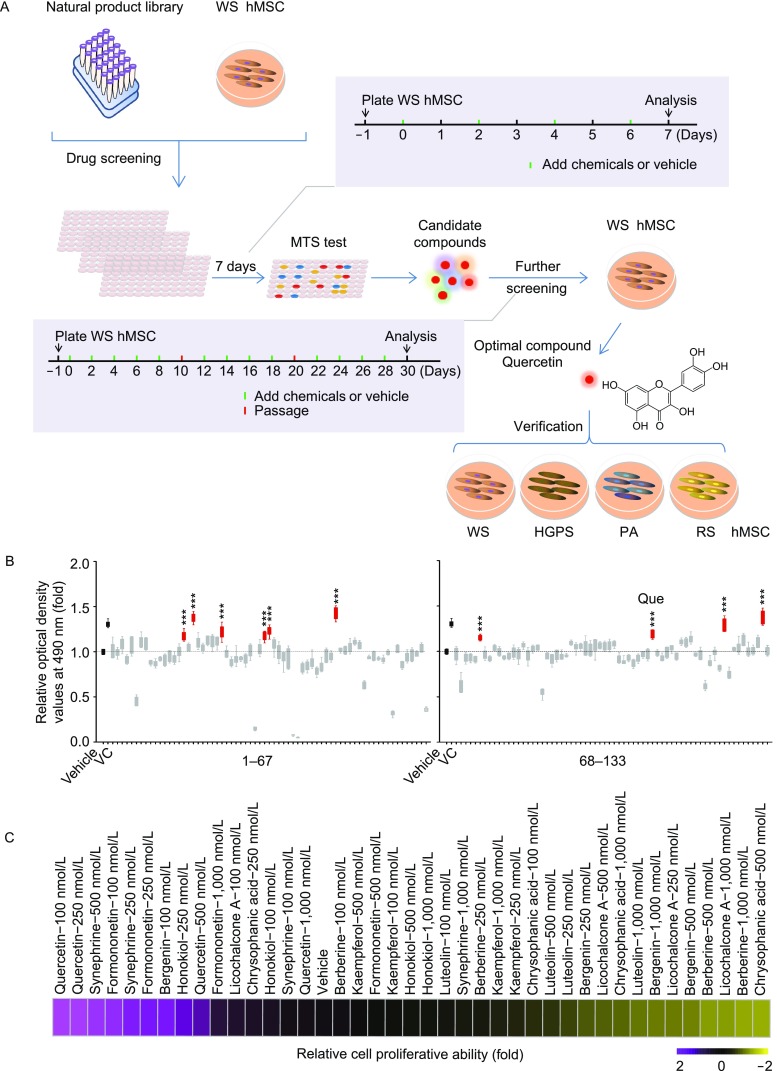
Screening of a natural product library in WS hMSCs. (A) Schematics of the screening strategy of a natural product library for geroprotective drugs. (B) Results from MTS assay highlighting ten candidate compounds (*n *= 5). ***P < 0.001 vs. vehicle. (C) Relative cell proliferative abilities upon the treatment of the ten candidate compounds at indicated concentrations (*n *= 3)

We have recently established a platform of Werner syndrome (WS) and Hutchinson-Gilford progeria syndrome (HGPS) hMSCs for studying premature and physiological aging (Fang et al., 2018; Kubben et al., 2016; Li et al., 2016b; Wu et al., 2018; Yang et al., 2017; Zhang et al., 2015). Here, we took advantage of this platform and screened for natural product compounds capable of alleviating premature senescence (Fig. 1A). A total of 133 natural products (Table S1) were evaluated using WS hMSCs through a short-term treatment of each compound at 1 μmol/L for seven days. The effect on cell proliferation by each compound was evaluated by 3-(4,5-dimethylthiazol-2-yl)-5-(3-carboxymethoxyphenyl)-2-(4-sulfophenyl)-2H-tetrazolium, inner salt (MTS) assay and ten compounds including quercetin dihydrate (referred to as quercetin, Que), chrysophanic acid, licochalcone A, luteolin, formononetin, honokiol, berberine HCl, kaempferol, bergenin and synephrine (Fig. 1B and Table S1) with at least 15% higher cell proliferative ability than that of the vehicle control (DMSO) were identified. These ten compounds were further evaluated through a long-term treatment at different concentrations for 30 days (Fig. 1A–C). Que was selected due to its leading effects for further studies.

### Dose optimization of Que

**Figure 2:**
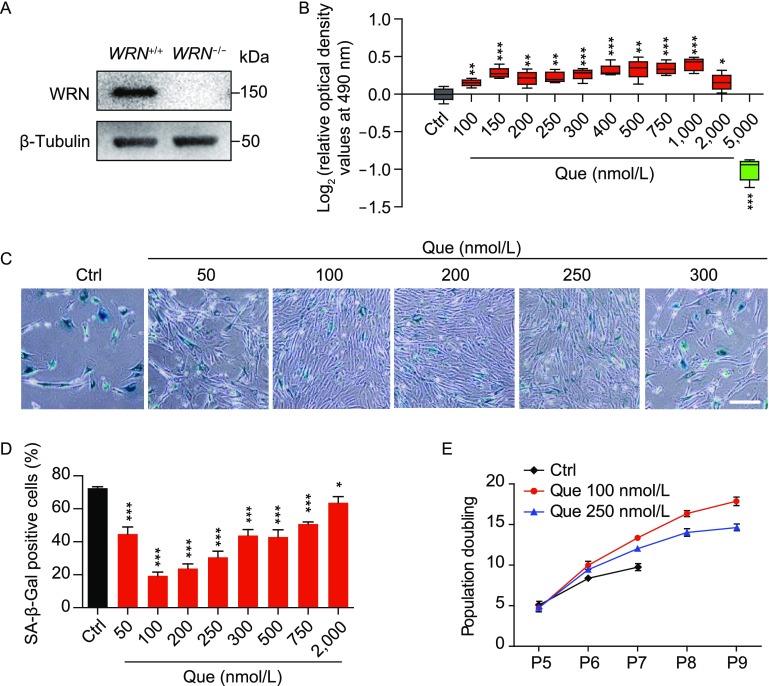
Identification of the optimal concentration of Que for alleviating aging and promoting proliferation of WS hMSCs. (A) Immunoblotting analysis of WRN protein in WT and WS hMSCs (passage 5). β-Tubulin was used as the loading control. (B) MTS assay of WS hMSCs (passage 5) after 7-day treatment of Que at a series of concentrations (*n* = 5). *P < 0.05 vs. Ctrl; **P < 0.01 vs. Ctrl; ***P < 0.001 vs. Ctrl. (C) Analysis of SA-β-Gal activity in vehicle- (Ctrl) and Que-treated WS hMSCs (passage 7). Representative images of SA-β-Gal staining. Scale bar, 100 μm. (D) Frequency of SA-β-Gal positive cells (*n* = 3). *P < 0.05 vs. Ctrl; ***P < 0.001 vs. Ctrl. (E) Accumulative growth curve showing the population doubling of vehicle- and Que-treated hMSCs (*n* = 3)

To identify the optimal concentration of Que in WS hMSCs (Fig. 2A), we first treated mid-passage (passage 5) WS hMSCs with different concentrations of Que for seven days. Then cell proliferative abilities were evaluated by MTS assay, showing the beneficial effects of Que at concentrations ranging from 100 nmol/L to 2 μmol/L (Fig. 2B). Furthermore, we treated WS hMSCs (passage 5) with different concentrations of Que for a longer term (30 days). Assessment of senescence-associated-β-galactosidase (SA-β-Gal) and cellular proliferation ability of WS hMSCs at passage 7 identified the optimal concentration of Que at 100 nmol/L for mechanistic studies (Fig. 2C–E).

### Que alleviated senescence and promoted cell self-renewal and differentiation in WS hMSCs

**Figure 3:**
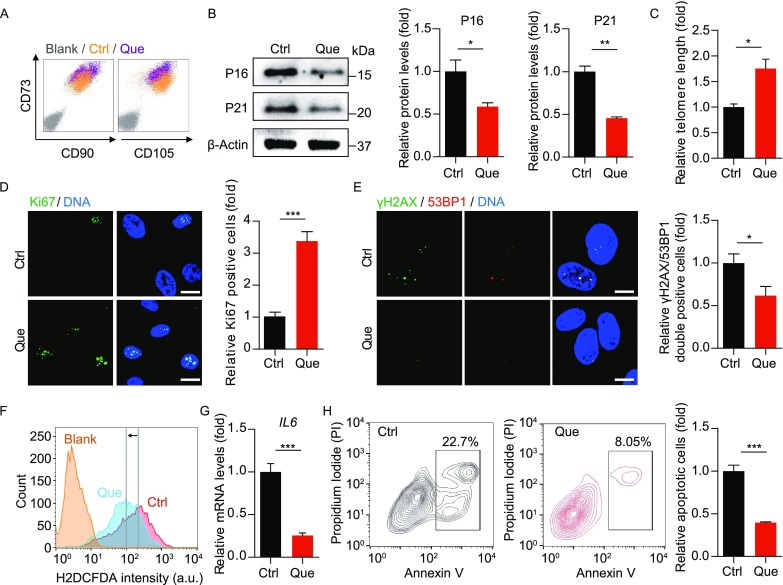
Que alleviated senescence and promoted self-renewal of WS hMSCs. (A) FACS analysis of hMSC-specific markers (CD73, CD90 and CD105) in vehicle- and Que-treated WS hMSCs. (B) Immunoblotting analysis of aging-related markers P16 and P21 in vehicle- and Que-treated WS hMSCs (passage 7). β-Actin was used as the loading control. Data are shown as mean ± SEM (*n* = 3). *P < 0.05 vs. Ctrl; **P < 0.01 vs. Ctrl. (C) Quantitative PCR analysis of telomere length of vehicle- and Que-treated WS hMSCs (passage 7). Data are shown as mean ± SEM (*n* = 3). *P < 0.05 vs. Ctrl. (D) Immunostaining of Ki67 in vehicle- and Que-treated WS hMSCs (passage 7), Scale bar, 25 μm. Data are shown as mean ± SEM (cell number ≥ 300). ***P < 0.001 vs. Ctrl. (E) Immunostaining of γ-H2AX and 53BP1 in vehicle- and Que-treated WS hMSCs (passage 7), Scale bar, 10 μm. The percentage of γ-H2AX/53BP1-double positive cells are shown as mean ± SEM (cell number ≥ 300). *P < 0.05 vs. Ctrl. (F) FACS measurement of reactive oxygen species (ROS) by H2DCFDA in vehicle- and Que-treated WS hMSCs (passage 7). a.u., arbitrary unit. (G) RT-qPCR analysis of *IL6* mRNA levels in vehicle- and Que-treated WS hMSCs (passage 7). Data are shown as mean ± SEM (*n* = 3). ***P < 0.001 vs. Ctrl. (H) Cell apoptosis assay in vehicle- and Que-treated WS hMSCs (passage 7). Quantitative data on the right are presented as mean ± SEM (*n* = 3). ***P < 0.001 vs. Ctrl

**Figure 4:**
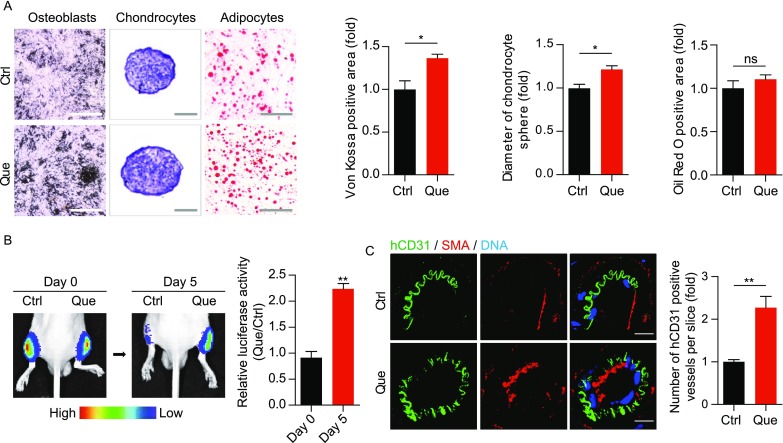
Que promoted cellular differentiation and vasculogenesis of WS hMSCs. (A) Characterization of osteogenesis, chondrogenesis and adipogenesis potentials of vehicle- and Que-treated WS hMSCs (passage 7) stained by Von Kossa (*n* = 3 independent experiments), toluidine blue O (*n* = 15 spheres) and oil red O (*n* = 3 independent experiments), respectively. Data are presented as mean ± SEM. *P < 0.05 vs. Ctrl; ns, not significant. Scale bar, 100 μm. (B) *In vivo* hMSC implantation assay with vehicle- and Que-treated WS hMSCs. Quantitative data on the right are presented as mean ± SEM. **P < 0.01 vs. Ctrl (*n* = 6). (C) Fat pad transplantation with vehicle- and Que-treated WS hMSCs (passage 7). Left: representative immunofluorescent images showing neovascularization; right: the number of hCD31-positive vessels calculated based on 20 slices from inconsecutive frozen sections from six mice each group. **P < 0.01 vs. Ctrl. Scale bar, 10 μm

Que-treated WS hMSCs were able to express hMSC-specific markers including CD73, CD90 and CD105 (Fig. 3A). Que alleviated the senescent phenotypes of late-passage WS hMSCs, evidenced by decreased senescent markers P16 and P21 (Fig. 3B), increased telomere length (Fig. 3C), increased cell proliferative potential by Ki67 staining (Fig. 3D), and decreased DNA damage response markers γ-H2AX and 53BP1 (Fig. 3E). Reactive oxygen species (ROS) production (Labbé et al., 2010) (Fig. 3F), mRNA levels of proinflammatory cytokine *IL-6* (Fig. 3G) and cell apoptosis (Fig. 3H) were each suppressed in Que-treated WS hMSCs. In addition, Que-treated WS hMSCs maintained the ability to differentiate towards multiple mesodermal lineages, with enhanced potentials towards osteogenesis and chondrogenesis (Fig. 4A). Furthermore, Que attenuated the *in vivo* decay of WS hMSCs implanted into the tibialis anterior muscles of nude mice (Fig. 4B) and enhanced the vasculogenic ability of WS hMSCs implanted into the fat pads of non-obese diabetic (NOD) scid mice (Fig. 4C). Taken together, these data suggest that Que alleviated cellular senescence and promoted self-renewal and differentiation abilities in WS hMSCs.

### Que restored the heterochromatin architecture in WS hMSCs

**Figure 5:**
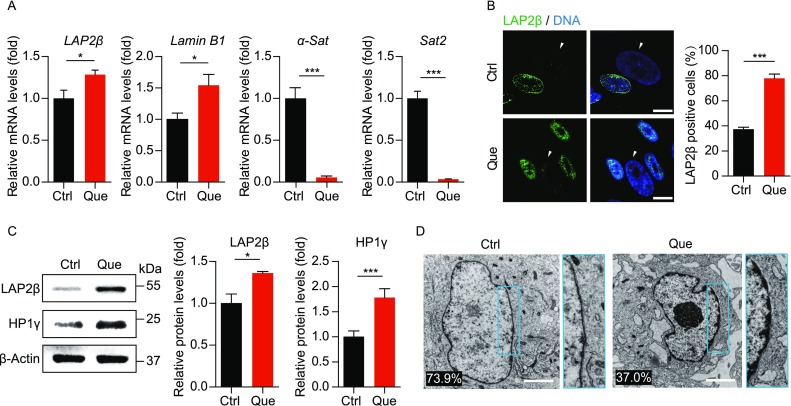
Que rejuvenated epigenetic features of WS hMSCs. (A) RT-qPCR analysis of *LAP2β*, *Lamin B1*, *α-Sat* and *Sat2* mRNA levels in vehicle- and Que-treated WS hMSCs (passage 7). Data are shown as mean ± SEM (*n* = 3). *P < 0.05 vs. Ctrl; ***P < 0.001 vs. Ctrl. (B) Immunostaining of LAP2β in vehicle- and Que-treated WS hMSCs. White arrows indicate LAP2β-negative nuclei. Quantitative data on the right are shown as mean ± SEM (cell number ≥ 300). ***P < 0.001 vs. Ctrl. Scale bar, 10 μm. (C) Immunoblotting analysis of LAP2β and HP1γ in vehicle- and Que-treated WS hMSCs (passage 7). β-Actin was used as the loading control. Quantitative data are shown as mean ± SEM (*n* = 3). *P < 0.05 vs. Ctrl; ***P < 0.001 vs. Ctrl. (D) Visualization of heterochromatin architecture of vehicle- and Que-treated WS hMSCs (passage 7) by transmission electron microscope (TEM). High-magnification views of heterochromatin around nuclear envelope are shown on the right. The percentage of cells with sparse heterochromatin at nuclear periphery is indicated at the lower left corner of each representative image. Cell number > 100. Scale bar, 2 μm

Because Werner syndrome is caused by mutations in *WRN* gene that encodes a RecQ DNA helicase important to DNA replication and chromatin maintenance (Li et al., 2016b; Wu et al., 2018; Yu et al., 1996; Zhang et al., 2015), genomic instability and heterochromatin disorganization manifest in WS pathogenesis (Li et al., 2016b; Murfuni et al., 2012; Ren et al., 2017a; Ren et al., 2011; Seki et al., 2008; Shamanna et al., 2017; Wu et al., 2018; Zhang et al., 2015). Upon Que treatment, the transcription of pericentromeric repetitive sequences including α-satellite (*α-Sat*) and satellite 2 (*Sat2*) were downregulated, whereas nuclear envelope proteins Lamin B1 and LAP2β were transcriptionally upregulated (Fig. 5A). Likewise, the protein levels of LAP2β and heterochromatin marker HP1γ were also increased (Fig. 5B and 5C). Lastly, direct visualization of nuclear structure by transmission electron microscope (TEM) revealed heterochromatin loss reduced from 73.9% to 37.0% in WS hMSCs upon Que treatment (Fig. 5D). Taken together, these data suggest that Que repressed heterochromatin disorganization in premature aging hMSCs.

### Transcriptome analysis revealed rejuvenation of WS hMSCs by Que

**Figure 6:**
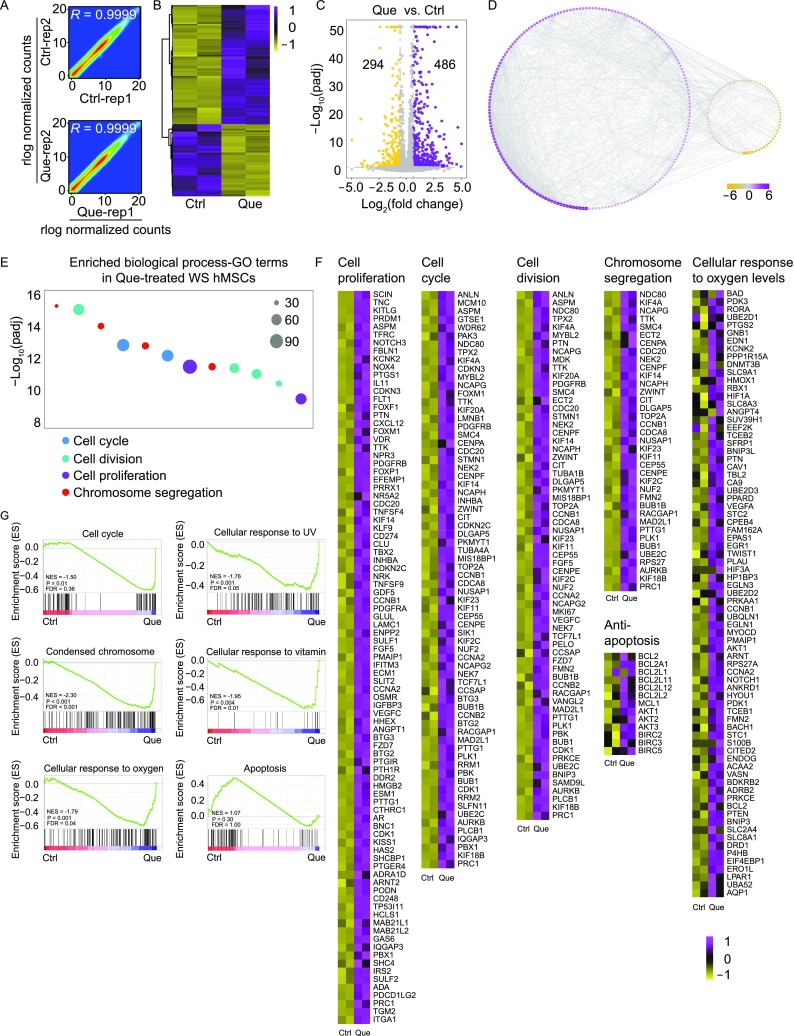
Gene expression profile analysis of vehicle- and Que-treated WS hMSCs. (A) Scatter plots showing the correlation between replicates of vehicle- (Ctrl) and Que-treated WS hMSCs. (B) Heatmap illustrating differentially expressed genes in Que-treated WS hMSCs compared to Ctrl (passage 7). (C) Volcano plot showing the number of upregulated and downregulated genes upon Que treatment. (D) Interaction network showing protein-protein interactions (PPI) of differentially expressed genes with |Log_2_(fold change)| > 1 and interaction score > 0.9 based on the STRING database. Purple indicates upregulation and yellow indicates downregulation; node size indicates the degree of upregulation and downregulation. (E) GO enrichment analysis of upregulated genes in Que-treated hMSCs. (F) Heatmaps showing the transcriptional levels of genes enriched in various gene terms. (G) Gene set enrichment analysis (GSEA) plots showing representative gene terms related to the functions of Que. The plots were based on the results from KEGG analysis for the cell cycle and apoptosis pathways and GO analysis for the condensed chromosome and cellular response to oxygen, vitamin and UV

To uncover the molecular mechanisms by which Que rejuvenated WS hMSCs, we performed genome-wide RNA sequencing (RNA-seq). The correlation coefficients between replicates were 0.9999 in vehicle- (Ctrl) and Que-treated WS hMSCs, indicating that the RNA-seq data were of high reproducibility (Fig. 6A). A total of 486 upregulated genes and 294 downregulated genes were obtained in Que-treated WS hMSCs relative to Ctrl (|Log_1.5_(fold change)| > 1, adjust *P* value (padj) < 0.05) (Fig. 6B, 6C, Tables S2 and S3). Protein-protein interactions (PPI) network of differentially expressed genes was drawn based on the STRING database (Fig. 6D). Gene Ontology (GO) analysis revealed that the upregulated genes in Que-treated WS hMSCs were enriched in biological processes including cell cycle, cell division, chromosome segregation, and cell proliferation (Fig. 6E and 6F). Consistently, gene set enrichment analysis (GSEA) data revealed that Que-treated WS hMSCs were enriched in GO terms including cell cycle, condensed chromosome, as well as cellular response to oxygen, UV and vitamin (Fig. 6F and 6G). On the contrary, vehicle-treated WS hMSCs were enriched in apoptosis pathway (Fig. 6G). The anti-apoptotic function of Que was further evidenced by the increased transcriptional levels of typical anti-apoptotic genes including genes in *BCL2*, *IAP* and *AKT* families (Fig. 6F), consistent with the aforementioned results showing decreased cell apoptosis in Que-treated WS hMSCs.

**Figure 7:**
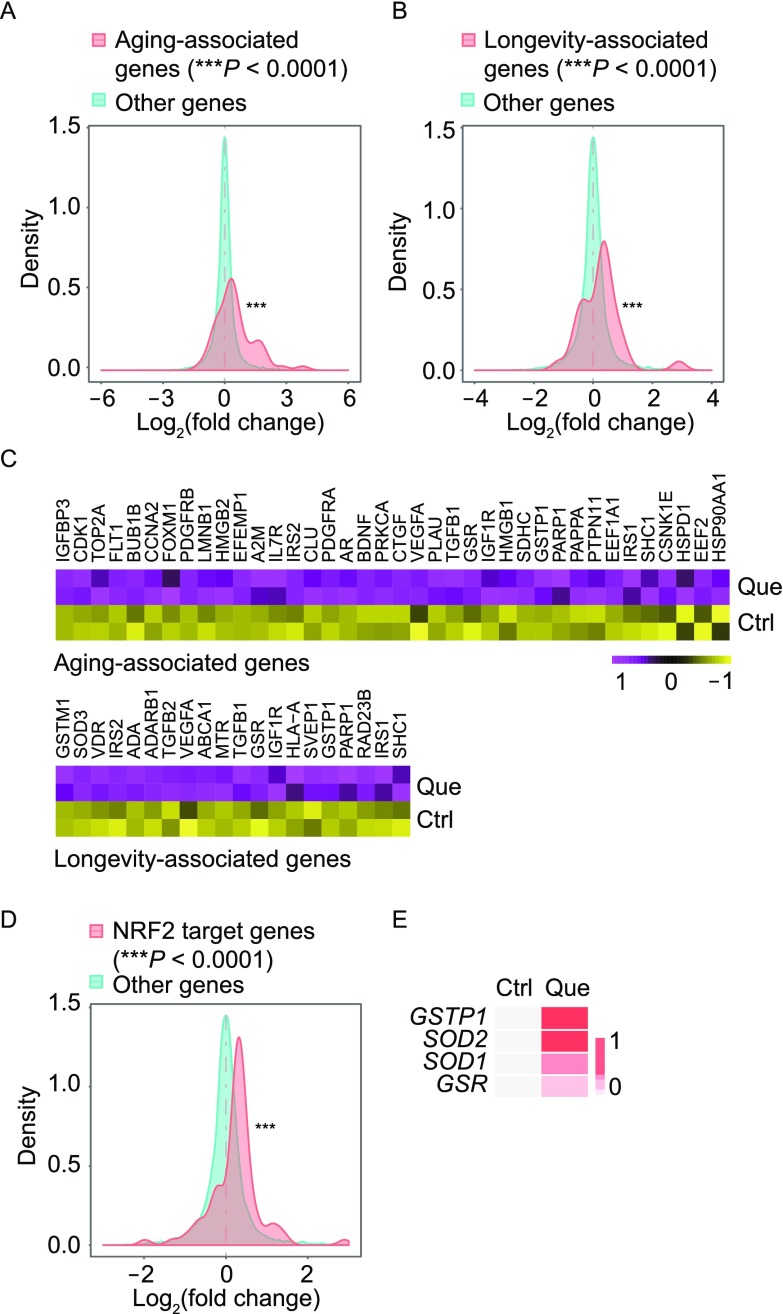
Gene expression analysis revealed the regulation of aging/longevity-related genes, and NRF2 target genes upon Que treatment. (A) Density plot showing Log_2_(fold change) of mRNA expression levels between Que-treated and vehicle-treated (Ctrl) WS hMSCs for aging-associated genes. A rightward shift (***P < 0.0001) indicates increased frequency of genes upregulated upon Que treatment. (B) Density plot showing Log_2_(fold change) of mRNA expression levels between Que-treated and Ctrl WS hMSCs for longevity-associated genes. (C) Heatmap showing upregulated genes associated with aging and longevity upon Que treatment. (D) Density plot showing Log_2_(fold change) of mRNA expression levels between Que-treated and Ctrl WS hMSCs for NRF2 target genes. (E) RT-qPCR analysis of the transcriptional levels of NRF2 target genes in vehicle- and Que-treated WS hMSCs (*n* = 3) (passage 7)

To investigate whether Que was directly involved in the regulation of aging biological process, we checked the transcriptional levels of aging and longevity-associated genes annotated by the Human Ageing Genomic Resources (HAGR) database. Gene expression analysis showed that aging and longevity-associated genes were upregulated (*P* < 0.0001) upon Que treatment (Fig. 7A–C). Of note, a series of anti-oxidative genes including *GSTP1*, *GSR* and *GSTM1* were upregulated (Fig. 7C). Given that all these genes were NRF2 responsive genes, we tested whether the anti-oxidative function of Que was NRF2-dependent by evaluating the expression levels of NRF2 target genes. The results showed that NRF2 target genes were upregulated (*P* < 0.0001) upon Que treatment (Fig. 7D). The increased expression levels of *GSTP1*, *GSR*, *SOD1* and *SOD2* in WS hMSCs upon Que treatment was reconfirmed by RT-qPCR analysis (Fig. 7E). Taken together, these data suggest that Que rejuvenated WS hMSCs via the regulation of multiple cellular processes including promoting cell cycle, condensing chromosome, and enhancing anti-oxidation.

### Transcriptomics comparison of the geroprotective effects of Que and VC in WS hMSCs

**Figure 8:**
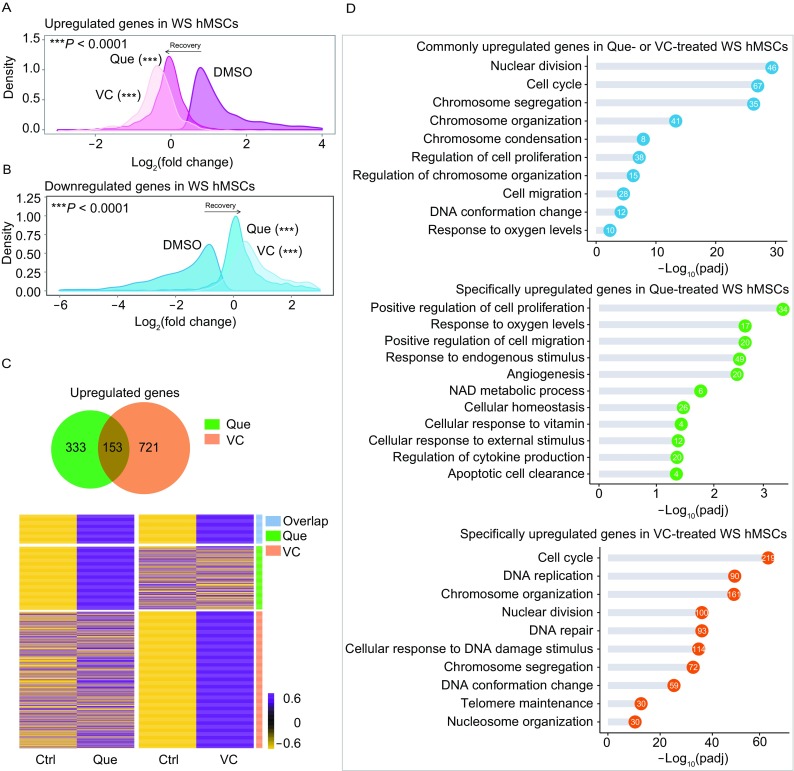
Comparative analysis of the RNA-seq data for Que- and VC-treated WS hMSCs. (A) Density plot showing upregulated genes in WS hMSCs restored upon Que or VC treatment. (B) Density plot showing downregulated genes in WS hMSCs restored upon Que or VC treatment. (C) Venn diagram and heatmap comparing upregulated genes in Que- or VC-treated WS hMSCs. (D) GO enrichment analysis for commonly upregulated genes in Que- or VC-treated WS hMSCs (top), genes only upregulated in Que-treated WS hMSCs (middle), and genes only upregulated in VC-treated WS hMSCs (bottom). The number of enriched genes in each GO term is shown in the circles

We have previously reported that Vitamin C (VC) is a rejuvenating factor for WS hMSCs by altering the expression of genes involved in chromatin condensation, cell cycle regulation, DNA replication, and DNA damage repair pathways (Li et al., 2016b). Here, we performed a conjoint analysis of RNA-seq data for Que- and VC-treated WS hMSCs to compare and contrast the underlying mechanisms of their geroprotective effects. Firstly, the upregulated and downregulated genes in WS hMSCs compared to WT hMSCs were reversed by either Que or VC treatment (Fig. 8A and 8B). We further analyzed the upregulated genes in Que- and VC-treated WS hMSCs. Among these genes, 333 were specific to Que treatment and 721 to VC treatment, with 153 to both Que and VC treatment (Fig. 8C). Notably, the commonly upregulated genes were enriched in biological process GO terms related to cell cycle, chromatin condensation and anti-oxidation, whereas Que-specific upregulated genes were mostly enriched in response to stimulus and cell homeostasis and the VC-specific ones in DNA replication, DNA repair and telomere maintenance (Fig. 8D). Thus, these data reveal the major commonalities in the mechanisms by which Que and VC alleviated aging phenotypes in WS hMSCs.

### Que attenuated senescence in Hutchinson-Gilford progeria syndrome hMSCs

**Figure 9:**
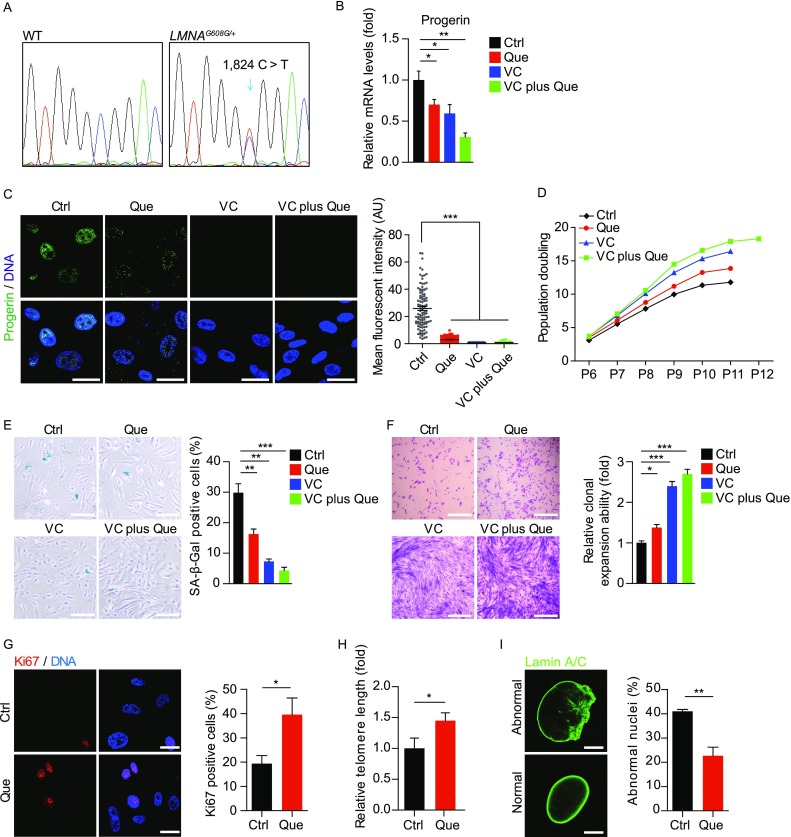
Que alleviated senescence in HGPS hMSCs with a synergistic effect with VC. (A) Confirmation of the heterozygous mutation of *LMNA* by DNA sequencing. (B) RT-qPCR analysis of progerin mRNA expression in vehicle-, Que-, VC- and VC plus Que-treated HGPS hMSCs (passage 10). Data are shown as mean ± SEM (*n* = 3). *P < 0.05 vs. Ctrl; **P < 0.01 vs. Ctrl. (C) Immunostaining of progerin in vehicle-, Que-, VC- and VC plus Que-treated HGPS hMSCs (passage 10), Scale bar, 25 μm. Mean fluorescence intensity on the right is shown as mean ± SEM (cell number ≥ 300). ***P < 0.001 vs. Ctrl. (D) Accumulative growth curve showing the population doubling of HGPS hMSCs upon the treatment of vehicle, Que, VC and VC plus Que (*n* = 3). (E) Analysis of SA-β-Gal activity in vehicle-, Que-, VC- and VC plus Que-treated HGPS hMSCs. Left, representative images of SA-β-Gal staining (passage 10); right, frequency of SA-β-Gal-positive cells. Data are shown as mean ± SEM (*n* = 3). **P < 0.01 vs. Ctrl; ***P < 0.001 vs. Ctrl. Scale bar, 100 μm. (F) Clonal expansion ability of vehicle-, Que-, VC- and VC plus Que-treated HGPS hMSCs (passage 8) stained by crystal violet. Quantitative data are shown as mean ± SEM (*n* = 3). *P < 0.05 vs. Ctrl; ***P < 0.001 vs. Ctrl. Scale bar, 100 μm. (G) Immunostaining of Ki67 in vehicle- and Que-treated HGPS hMSCs (passage 10). Scale bar, 25 μm. Ki67-positive cells are shown as mean ± SEM (cell number ≥ 300). *P < 0.05 vs. Ctrl. (H) Quantitative PCR analysis of telomere length in vehicle- and Que-treated HGPS hMSCs (passage 10). Data are shown as mean ± SEM (*n* = 3). *P < 0.05 vs. Ctrl. (I) Immunostaining of Lamin A/C in vehicle- and Que-treated HGPS hMSCs (passage 10). Quantifications of abnormal nuclei of HGPS hMSCs on the right are shown as mean ± SEM (*n* ≥ 300). **P < 0.01 vs. Ctrl. Scale bar, 10 μm

Besides WS, Hutchinson-Gilford progeria syndrome (HGPS) hMSCs are another premature senescent stem cell model we recently established by introducing heterozygous *LMNA*^*G608G*/+^ mutation into human embryonic stem cells (hESCs) followed by directed differentiation into hMSCs (Kubben et al., 2016; Wu et al., 2018). DNA sequencing confirmed the presence of heterozygous mutation of *LMNA*^G608G/+^ in HGPS hMSCs (Fig. 9A). The mRNA level and immunostaining intensity of progerin, which is the resultant truncated protein product of mutated *LMNA*, were decreased in Que-, VC- and VC plus Que-treated HGPS hMSCs (Fig. 9B and 9C). The alleviation of cellular senescence by Que, VC, or VC plus Que in HGPS hMSCs was further evidenced by decreased population doubling time (Fig. 9D), decreased SA-β-Gal positivity (Fig. 9E), and increased clonal expansion and proliferative ability (Fig. 9F–H). In addition, the percentage of abnormal nuclei, a cellular hallmark of HGPS, decreased in HGPS hMSCs upon Que treatment (Fig. 9I). Taken together, these data indicate that Que attenuated accelerated senescence in HGPS hMSCs.

### Que ameliorated senescent phenotypes in physiological-aging hMSCs

**Figure 10:**
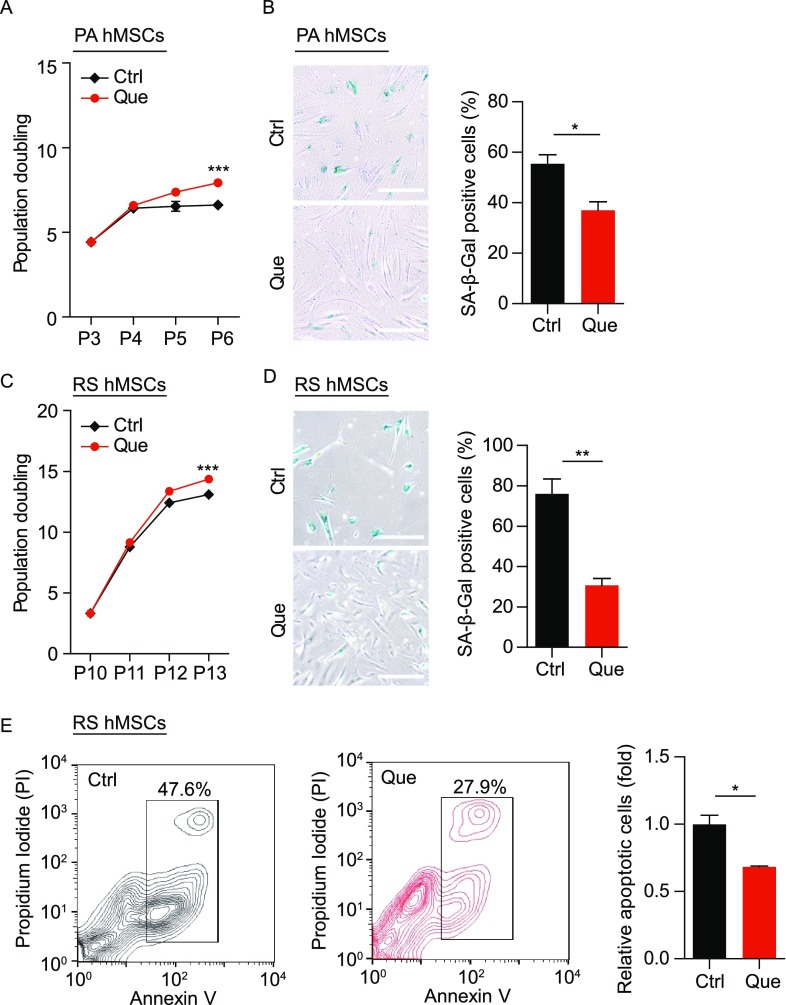
Que alleviated senescent phenotypes in physiological-aging (PA) hMSCs and replicative-senescent (RS) hMSCs. (A) Accumulative growth curve showing the population doubling of vehicle- and Que-treated PA hMSCs (*n* = 3). ***P < 0.001 vs. Ctrl. (B) Analysis of SA-β-Gal activity in vehicle- and Que-treated PA hMSCs (passage 5). Left, representative images of SA-β-Gal staining; right, frequency of SA-β-Gal-positive cells. Data are shown as mean ± SEM (*n* = 3). *P < 0.05 vs. Ctrl. Scale bar, 100 μm. (C) Accumulative growth curve showing the population doubling of vehicle- and Que-treated RS hMSCs (*n* = 3). ***P < 0.001 vs. Ctrl. (D) Analysis of SA-β-Gal activity in vehicle- and Que-treated RS hMSCs (passage 13). Left, representative images of SA-β-Gal staining; right, frequency of SA-β-Gal-positive cells. Data are shown as mean ± SEM (*n* = 3). **P < 0.01 vs. Ctrl. Scale bar, 100 μm. (E) Cell apoptosis assay in vehicle- and Que-treated RS hMSCs (passage 13). Quantitative data on the right are shown as mean ± SEM (*n* = 3). *P < 0.05 vs. Ctrl

We further examined the geroprotective effects of Que in physiological-aging (PA) wild-type hMSCs from a 56-year individual and replicative-senescent (RS) wild-type hMSCs differentiated from hESCs. Decreased population doubling time and SA-β-Gal positivity revealed enhanced self-renewal and suppressed senescence in PA and RS hMSCs upon Que treatment, respectively (Fig. 10A–D). In addition, cell apoptosis was alleviated in Que-treated RS hMSCs (Fig. 10E). These results suggest that besides progeria cells, Que also exerted geroprotective effects against physiological senescence in hMSCs.

## Discussion

**Figure 11:**
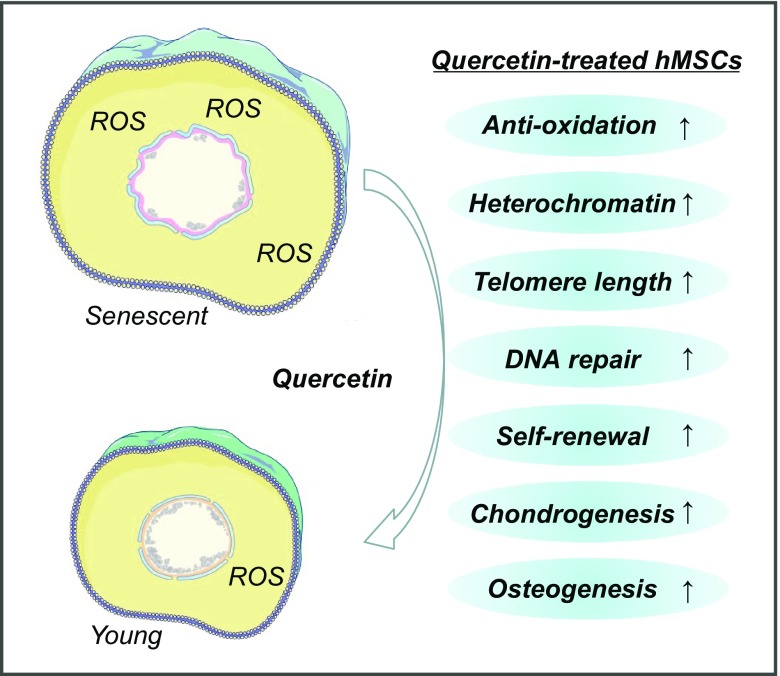
A proposed model illustrating the geroprotective effects of Que in hMSCs

Discovery of compounds that attenuate senescent phenotypes can lead to therapeutic advances that ameliorate aging-related pathologies (Chang et al., 2016; Kreienkamp et al., 2016; Li et al., 2016b). Here, by employing a platform with premature aging Werner syndrome (WS) hMSCs for the screening of effective compounds against cellular senescent phenotypes, we have identified ten candidate natural products, including quercetin dihydrate, chrysophanic acid, licochalcone A, luteolin, formononetin, honokiol, berberine HCl, kaempferol, bergenin and synephrine. Except Que and berberine HCl (Xu et al., 2017), the other eight compounds have never been implicated in premature aging-related studies, thus greatly broadening our view of geroprotective drug candidates. Further investigation revealed the mechanisms by which the top hit, Que exhibited beneficial effects in attenuating hMSC senescence: (1) Que alleviated senescence by promoting stem cell self-renewal and restoring the heterochromatin architecture in WS hMSCs; (2) transcriptome analysis supported that Que improved the “healthspan” of WS hMSCs; (3) Que and VC exerted geroprotective effects through overlapping mechanisms; (4) Que also alleviated cellular senescent phenotypes in HGPS and physiological-aging hMSCs (Fig. 11). For the first time, Que was identified as a geroprotective agent against both premature and physiological human aging, supporting the role of Que as a novel therapeutic option for treating premature aging and promoting healthy aging. Additionally, the generalized effectiveness of Que verifies the efficacy of WS hMSCs as a powerful model for geroprotective drug screening.

Que is a polyphenol derived from plants. In *C*. *elegans*, Que extends lifespan by 15% due to its antioxidant capacity indirectly via the activation of DAF-16 (homologue to mammalian FoxO proteins) (Kampkötter et al., 2008). Que is also reported to rejuvenate senescent primary human fibroblasts (Chondrogianni et al., 2010). Prior to our study, the effect of Que has not been evaluated in progeroid syndromes such as WS and HGPS. For the first time, we uncovered a geroprotective role of Que in human accelerated aging hMSCs. Que-treated WS hMSCs demonstrated improved differentiation potentials into osteoblasts and chondrocytes and enhanced *in vivo* vasculogenesis ability, implying potential therapeutic promises of Que with regard to symptoms such as osteoporosis and atherosclerosis in WS and HGPS patients. Of particular, we observed that Que effectively decreased progerin in HGPS hMSCs. Thus, our studies support Que as a potential therapeutic agent for treating WS and HGPS in clinic (Harhouri et al., 2018). Still, the *in vivo* effects of Que on rodents or non-human primates need further investigation.

The effects of high-dose Que have been previously evaluated in human cells and mice. The combination of dasatinib and high-dose of Que (50 mg/kg) effectively eliminates senescent cells via induced apoptosis and thus alleviated senescence-related phenotypes in mice (Zhu et al., 2015). In non-alcoholic fatty liver disease (NAFLD) mice, the combination of dasatinib and Que (20 mg/kg) eliminates senescent cells for the reduction of overall hepatic steatosis (Ogrodnik et al., 2017). In senescent human preadipocytes and umbilical vein endothelial cells (HUVECs), high-dose Que (optimal concentrations at 10 and 20 μmol/L, respectively) induces apoptotic cell death. By contrast, we used Que at 100 nmol/L in WS, HGPS, physiological-aging hMSCs, which effectively alleviated cellular senescence without inducing cell death, indicative of a much safer therapeutic dose potentially applicable to stimulating hMSC activity.

To further determine the molecular mechanisms of Que, we performed RNA-seq analysis and revealed that the upregulated genes in Que-treated WS hMSCs were primarily enriched in cell cycle, nuclear division and chromosome segregation pathways, consistent with the cellular phenotypes observed. Previous studies on Que have mainly focused on its anti-carcinogenic (Ansgar, 2013; Darband Saber et al., 2018), anti-inflammatory (Griffiths et al., 2016; Li et al., 2016a; Liu et al., 2017; Salvatore, 2010), anti-viral (Chiow et al., 2016; Li et al., 2016a), as well as anti-oxidant effects (Sohn et al., 2018). However, the mechanisms of Que against human stem cell aging remain to be elucidated. For the first time, we showed that Que alleviated hMSC aging at least partially via the reduction of ROS, and restoration of heterochromatin architecture, providing a new mechanism for Que-mediated geroprotection.

We have previously shown that VC alleviates aging defects in WS hMSCs by decreasing oxidative stress, preventing telomere attrition, suppressing excessive secretion of inflammatory factors, as well as reorganizing nuclear lamina and heterochromatin (Li et al., 2016b). Our comparative analysis of the RNA-seq data in WS hMSCs illustrated the mechanistic commonalities and differences of the geroprotective effects by Que and VC, with the commonly upregulated genes mainly enriched in gene terms related to cell cycle, chromatin condensation and anti-oxidation. Likewise, we observed that Que restored heterochromatin architecture and decreased ROS levels in WS hMSCs. It has been shown that increased ROS levels lead to heterochromatin disorganization. Therefore, in our study decreased ROS levels may also contribute to the restoration of heterochromatin architecture. In addition, we have recently demonstrated that, the premature aging phenotypes in HGPS hMSCs partially attributes to the repression of NRF2-mediated anti-oxidative response, whereas the reactivation of NRF2 reverses the nuclear defects in HGPS cells and restores their *in vivo* viability in mice (Fang et al., 2018; Kubben et al., 2016). Interestingly, Que is reported as an activator of NRF2-mediated anti-oxidative pathway (Bahar et al., 2017; Dai et al., 2018; Gao et al., 2018; Kim et al., 2018). Consistently, by RNA-seq analysis we identified that Que upregulated a series of anti-oxidative genes including *GSTP1*, *GSR* and *GSTM1*, all of which are NRF2 targets, indicating that Que may exert anti-oxidative effect via the activation of NRF2. Furthermore, despite of the multiple mechanistic commonalities of the geroprotective effects, Que-specific upregulated genes were mostly enriched in response to stimulus and cell homeostasis and the VC-specific ones in DNA replication, DNA repair and telomere maintenance, raising the possibility that Que and VC in combination might exhibit a synergistic geroprotective effect.

In this study, we showed that Que and VC exerted cooperative effects attenuating accelerated senescence in HGPS hMSCs. Besides VC, other geroprotective chemicals could also strengthen the biological activity of Que. For example, resveratrol and Que exhibit synergistic effects in the restoration of genes in pathways of glucose/lipid metabolism, liver function, cardiovascular system, and inflammation/immunity in mice fed with high-fat diet (Zhou et al., 2012). Moving forward, how to maximize the geroprotective effects of Que by combining with other components awaits further investigation.

In summary, we screened a natural product library in WS hMSCs and identified Que as a geroprotective agent against premature and physiological human aging through detailed mechanistic studies and multi-layer evaluations in WS, HGPS and physiological-aging hMSCs. We have also elucidated the transcriptional commonalities and differences in the geroprotective effects by Que and VC in WS hMSCs. In addition, given the multi-differentiation potentials of human pluripotent stem cells (hPSCs) and hMSCs, our platform with WS and HGPS hPSCs, hMSCs, and their derivatives provides a powerful tool for mechanistic studies and drug screening in various cell types, such as cardiovascular cells that are widely affected in WS and/or HGPS syndromes.

## Materials and methods

### Cell culture and differentiation

*WRN*^−/−^ hMSCs and *LMNA*^G608G/+^ hMSCs were obtained via the directed differentiation of *WRN*^−/−^ hESCs and *LMNA*^*G608G*/+^ hESCs that we previously established (Wu et al., 2018; Zhang et al., 2015). hESCs were maintained on mitomycin C-inactivated mouse embryonic fibroblast (MEF) feeder in hESC culture medium containing 80% DMEM/F12 (Gibco), 20% Knockout Serum Replacement (Gibco), 10 ng/mL FGF2 (Joint Protein Central), 0.1 mmol/L non-essential amino acids (NEAA, Gibco), 2 mmol/L GlutaMAX (Gibco) and 55 μmol/L β-mercaptoethanol (Invitrogen). hESCs used in this study were also cultured on Matrigel (BD Biosciences) with mTeSR medium (STEMCELL Technologies). The human MSCs were cultured in hMSC culture medium containing 90% α-MEM + Glutamax (Gibco), 10% fetal bovine serum (FBS, Gemcell, Lot A77E01F), 1 ng/mL FGF2 (Joint Protein Central) and 1% penicillin/streptomycin (Gibco).

Differentiation of hESCs into mesenchymal stem cells (MSCs) was performed as described previously (Duan et al., 2015; Fu et al., 2016; Wang et al., 2018a; Wang et al., 2018b; Zhang et al., 2015). Briefly, embryoid bodies were cultured in matrigel-coated plates with hMSC differentiation medium (α-MEM with GlutaMAX (Gibco), 10% FBS (Gemcell, Lot A77E01F), 10 ng/mL FGF2 (Joint Protein Central), 5 ng/mL TGFβ (Stemimmune LLC) and 1% penicillin/streptomycin (Gibco)). After about 10 days, the confluent MSC-like cells were passaged on gelatin-coated plate and cultured in hMSC culture medium containing 90% α-MEM with Glutamax (Gibco), 10% fetal bovine serum (FBS, Gemcell, Lot A77E01F), 1 ng/mL FGF2 (Joint Protein Central) and 1% penicillin/streptomycin (Gibco).

*In vitro* differentiation potentials of WS hMSCs into osteoblasts, chondrocytes and adipocytes (Pan et al., 2016; Wu et al., 2018; Zhang et al., 2015) were evaluated by Von Kossa (Abcam), toluidine blue (Sigma) and oil red O (Sigma) stainings, respectively.

### Preparation of physiological-aging hMSCs

Physiological-aging (PA) hMSCs were isolated from gingiva of a male individual at 56 years old. In details, a piece of tissue (sized approximately by 7 mm × 4 mm × 2 mm) was obtained from the gingival lamina propria of free gingiva after the removal of the gingival epithelium. The tissue was washed with PBS and minced into small pieces, followed by digestion with collagenase at 37 °C for 30 min. The hMSCs were collected upon centrifugation at 1,000 rpm for 5 min and were cultured in hMSC culture medium.

### Fluorescence-activated cell sorting (FACS)

The human MSCs were purified by sorting against CD73, CD90 and CD105 triple positive cells using FACS. In details, hMSCs were digested using TrypLE Express (Gibco) and washed twice by PBS. Cells were incubated with primary antibody diluted with 10% FBS in PBS for 30 min at room temperature and sorted by using flow cytometer (BD FACSAria IIIu).

### Screening of a natural product library

A total of 133 compounds of a natural product library (Selleck, L1400) were purchased and screened in WS hMSCs initially at 1 μmol/L. WS hMSCs (passage 5) were seeded into drug-stamped 96-well plates at a density of 3,000 cells per well. Culture medium with different kinds of compounds at 1 μmol/L was changed every other day. Seven days after the drug treatment, cell proliferation was measured by MTS assay (Promega). Values of quintic treatment wells were averaged and normalized to quintic control wells.

The top candidate compounds were further screened by a 30-day treatment at four different concentrations (100, 250, 500 and 1,000 nmol/L). WS hMSCs (passage 5) were seeded into 6-well plates at a density of 30,000 cells per well. Culture medium with different kinds of candidate compounds was changed every other day. The relative cell proliferative abilities of WS hMSCs (passage 7) treated with each compound at different concentrations were analyzed at day 30.

### Determination of optimal Que concentration

WS hMSCs (passage 5) were seeded into 96-well plates at a density of 3,000 cells per well and treated with different concentrations of quercetin hydrate (Que) (TCI, P0042). Culture medium with Que was changed every other day. Seven days after the drug treatment, cell proliferation was measured by MTS assay (Promega). Values of quintic treatment wells were averaged and normalized to quintic control wells and processed by calculating the Log_2_ (Que/vehicle control).

### Senescence-associated β-galactosidase staining

Senescence-associated β-galactosidase (SA-β-Gal) staining was performed as described previously (Debacq-Chainiaux et al., 2009). Briefly, hMSCs were washed in PBS and fixed at room temperature for 3 min in 2% formaldehyde and 0.2% glutaraldehyde. Fixed cells were then stained with freshly prepared staining solution for SA-β-Gal activity at 37 °C overnight. Images were taken and the percentage of positive cells was calculated.

### Clonal expansion assay

Single-cell clonal expansion assay was carried out as described (Duan et al., 2015; Wu et al., 2018). Briefly, WS hMSCs were plated at a density of 2,000 cells per well in gelatin coated 12-well plate and treated with vehicle and Que. The cell density was calculated after crystal violet staining.

### Western blotting

1 × 10^6^ cells were collected and lysed with 100 μL RIPA buffer (0.1% SDS, 50 mmol/L Tris-HCl (pH 7.5), 1% NP-40, 0.5% sodium deoxycholate, 150 mmol/L NaCl) supplemented with NaVO_4_, NaF and the protease-inhibitor mixture (Roche). BCA kit (Thermo Fisher Scientific) was used for measurement of protein concentration. 20 μg proteins for each sample were separated by SDS-PAGE and transferred onto a PVDF membrane (Millipore). The membrane was incubated with primary antibodies and then HRP-conjugated secondary antibodies. The quantification was performed with Image Lab software for ChemiDoc XRS system (Bio-Rad).

### Quantitative real-time PCR (RT-qPCR)

Total RNAs were extracted using TRIzol Reagent (Invitrogen). 1–2 μg of total RNAs were converted to cDNA by using GoScript Reverse Transcription System (Promega). Quantitative real-time PCR was performed with iTaq Universal SYBR Green Super mix (Bio-Rad) on a CFX384 Real-Time PCR system (Bio-Rad). Data were normalized to *18S* rRNA transcript and calculated using the ΔΔCq method. Quantitative RT-PCR primers used were listed in Table S4.

### Immunofluorescence microscopy

Cells were prepared as previously reported (Wu et al., 2018). Briefly, hMSCs were seeded on microscope coverslips and fixed with 4% formaldehyde in PBS for 30 min, permeabilized with 0.4% Triton X-100 in PBS for 30 min, and blocked with 10% donkey serum in PBS for 1 h. The hMSCs on coverslips were incubated with primary antibodies (diluted with 1% donkey serum in PBS) at 4 °C overnight, and then incubated with fluorescence-labeled secondary antibodies (diluted with 1% donkey serum in PBS) at room temperature for 1 h. Hoechst 33342 (Invitrogen) was used to Stain nuclear DNA.

### *In vivo* stem cell retention analysis

WS hMSCs implantation was performed as previously described (Zhang et al., 2015). In brief, 5 × 10^5^ luciferase-expressing WS hMSCs were pretreated with vehicle or 100 nmol/L Que for 30 days and then implanted into the middle of the tibialis anterior muscle of immunodeficient mice. Five days after the implantation, mice were anaesthetized and injected with D-luciferin solution (Gold-Bio, luck-500). After 15 min the *in vivo* luciferase activity of each mouse was determined by the IVIS lumina system (PerkinElmer). Luminescence intensity was normalized to luciferase intensity of hMSCs right before the implantation. All the animal experiments performed in this study were approved by the Institute of Biophysics, Chinese Academy of Sciences.

### Fat pad vessel formation assay

Fat pad transplantation was performed as previously described (Yu et al., 2016). Freshly collected vehicle- or Que-treated WS hMSCs (passage 7) were resuspended in Matrigel mixture consisting of 50% Matrigel, 20% FBS in PBS, and 0.01% Trypan Blue (Sigma). 1.5 × 10^5^ cells in 15 μL volume was injected into the fat pads of 3-week-old female non-obese diabetic (NOD) scid mice. Fat pads were harvested 4 weeks post-transplantation for measuring donor-derived vessel regeneration by immunofluorescent staining.

### RNA library construction and RNA sequencing

WS hMSCs were treated with vehicle or Que from passage 5 and collected at passage 7 for RNA-seq analysis using Illumina sequencing platform. RNA sequencing libraries were prepared as previously reported (Li et al., 2016b; Zhang et al., 2015). Briefly, RNA integrity was examined by the Bioanalyzer 2100 system (Agilent Technologies). Sequencing libraries were constructed using NEBNext UltraTM RNA Library Prep Kit for Illumina (NEB) and sequenced on Illumina Hiseq X Ten platform.

### RNA-seq data processing

The pipeline of RNA-seq data processing has been described previously (Zhang et al., 2015). In brief, sequencing reads were trimmed and mapped to hg19 human genome using hisat2 software (v2.0.4) (Kim et al., 2015). The transcriptional expression level of each gene was counted by HTSeq (v0.6.1) (Anders et al., 2015). Differentially expressed genes (DEGs) were computed using DESeq2 at a cutoff adjust *P* value (Benjamini-Hochberg) less than 0.05 and |Log_1.5_(fold change)| more than 1 (Love et al., 2014). The correlation between replicates of each sample was evaluated by the Pearson correlation coefficient (R), which was based on DESeq2 regularized-logarithm (rlog) normalized read count. Gene Ontology (GO) and pathway enrichment analysis was conducted by ToppGene (Chen et al., 2009). Protein-protein interaction networks of differentially expressed genes was drawn based on the STRING database (Szklarczyk et al., 2017).

Transcription levels of aging- and longevity-associated genes as well as the NRF2 target genes were analyzed as below. The aging- and longevity-associated genes were identified according to the Human Ageing Genomic Resources (HAGR) database and NRF2 target genes from the C3 transcription factor targets in Molecular Signatures Database v6.0 (MSigDB, GSEA) with the addition of other NRF2 genes that have been previously reported (Kubben et al., 2016; Reszka et al., 2015; Tacutu et al., 2013). Genes with an adjust *P* value (Benjamini-Hochberg) less than 0.05 were analyzed and *P* values were calculated by Two-sample Kolmogorov-Smirnov test.

The RNA-seq data have been deposited to the NCBI Gene Expression Omnibus (GEO) database with accession number GSE116166.

### Antibodies

Primary antibodies for FACS were anti-CD73-PE (550741, 1:100), anti-CD90-FITC (555595, 1:200) from Biosciences and anti-CD105-APC (17-1057, 1:100) from eBioscience. Primary antibodies for Western blot were anti-WRN (sc-5629, 1:500), anti-β-Actin (sc-130301, 1:3,000), anti-β-Tubulin (sc-5274, 1:3,000) from Santa Cruz Biotechnology, anti-P21 (2947, 1:2,000), anti-HP1γ (2619, 1:1,000) from Cell Signaling Technology, anti-LAP2β (611000, 1:2,000) and anti-P16 (4828, 1:200) from BD Bioscience. Antibodies for immunofluorescent staining were anti-hSMA (ZM-0003) from ZSGB-Bio, anti-Progerin (sc-81611, 1:50), anti-Lamin A/C (sc-7293, 1:200) from Santa Cruz Biotechnology, anti-HP1γ (2619, 1:500) from Cell Signaling Technology, anti-53BP1 (A300-273A, 1:500) from Bethyl Laboratories, anti-γ-H2AX (05-636, 1:500) from Millipore, anti-LAP2β (611000, 1:500), anti-hCD31 (555445, 1:200) from BD Bioscience, and anti-Ki67 (VP-RM04, 1:1,000) from Vector.

### Statistical analysis

Student’s *t*-test was used for statistical analysis. Data are presented as mean ± SEM. *P* < 0.05 is considered statistically significant.

## Electronic supplementary material

Below is the link to the electronic supplementary material.

Supplementary material 1 (PDF 86 kb)

## Abbreviations

hESCs: human embryonic stem cells
HGPS: Hutchinson-Gilford progeria syndrome
hMSCs: human mesenchymal stem cells
Que: Quercetin
MTS: 3-(4,5-dimethylthiazol-2-yl)-5-(3-carboxymethoxyphenyl)-2-(4-sulfophenyl)-2H-tetrazolium, inner salt
PA: physiological-aging
ROS: reactive oxygen species
RS: replicative-senescent
SA-β-Gal: senescence-associated-β-galactosidase
SASP: senescence-associated secretory phenotype
TEM: transmission electron microscope
VC: Vitamin C
WS: Werner syndrome
