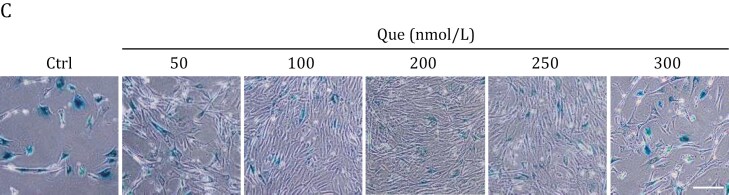# Correction to: Chemical screen identifies a geroprotective role of quercetin in premature aging

**DOI:** 10.1093/procel/pwaf023

**Published:** 2025-03-29

This is a **correction** to:

Lingling Geng, Zunpeng Liu, Weiqi Zhang, Wei Li, Zeming Wu, Wei Wang, Ruotong Ren, Yao Su, Peichang Wang, Liang Sun, Zhenyu Ju, Piu Chan, Moshi Song, Jing Qu, Guang-Hui Liu, Chemical screen identifies a geroprotective role of quercetin in premature aging, *Protein & Cell*, Volume 10, Issue 6, June 2019, Pages 417–435, https://doi.org/10.1007/s13238-018-0567-y

In the originally published version of this manuscript, there was an oversight in Figure 2C due to an image in the 200nmol/L group inadvertently sourced from a different experimental group. While this does not alter our conclusions or discussions, we are committed to upholding the highest publication standards and thus an updated Figure 2C has been supplied.

These details have been corrected only in this correction notice to preserve the published version of record.

On [date of resupply of the image], this correction notice was amended, since the version of Figure 2 it originally contained was not the corrected version. The figure has now been replaced with this corrected version.