# Supplementary material for: Chemical screen identifies a geroprotective role of quercetin in premature aging
Source: Protein Cell. 2018 Aug 1;10(6):417–35. doi: 10.1007/s13238-018-0567-y (PMC6538594; doi:10.1007/s13238-018-0567-y)
Supplement: Supplementary file 1 — Supplementary material 1 (PDF 86 kb) [file 13238_2018_567_MOESM1_ESM.pdf]

Table S1. List of compounds in the natural product library. The top ten candidate compounds are highlighted in red.

Table S2. Top 20 upregulated genes in Que-treated WS hMSCs.

Table S3. Top 20 downregulated genes in Que-treated WS hMSCs.

Table S4. Quantitative RT-PCR primers used.

Table S1 Natural product library

Number	Compound	Number	Compound
1	Xanthone	68	(+) -Usniacin
2	Gastrodin	69	Sclareolide
3	Quercetin	70	Tanshinone IIA
4	Isoliquiritigenin	71	Artesunate
5	Rotundine	72	Caffeic Acid
6	Piperlongumine	73	DL -Carnitine HCl
7	Yohimbine HCl	74	Gramine
8	Hematoxylin	75	Kaempferol
9	Sesamin	76	Morin Hydrate
10	Sophocarpine	77	Oleanolic Acid
11	Guanosine	78	Phlorizin
12	Abscisic Acid (Dormin）	79	3-Indolebutyric acid (IBA)
13	5-hydroxytryptophan (5-HTP)	80	Asiatic Acid
14	Hordenine	81	Chlorogenic Acid
15	Sorbitol	82	Emodin
16	Chrysophanic Acid	83	Gynostemma Extract
17	Inosine	84	Kinetin
18	Licochalcone A	85	Myricetin
19	Aloin	86	Oridoni n
20	Indirubin	87	Piperine
21	Naringenin	88	Shikimic Acid
22	Curcumol	89	Taxifolin (Dihydroquercetin)
23	Vanillin	90	4-Methylumbelliferone (4-MU)
24	Formononetin	91	Azomycin
25	Biochanin A	92	Chrysin
26	Lappaconite HBr	93	Enoxolone
27	Salidroside	94	Hesperetin
28	Astragaloside A	95	L-(+) -Rhamnose Monohydrate
29	N6 -methyladenosine (m6A)	96	Myricitrin
30	Xanthohumol	97	Orotic acid (6-Carboxyuracil)
31	Dioscin	98	Puerarin
32	L -carnitine	99	Silibinin
33	Honokiol	100	Tetrahydropapaverine HCl
34	Luteolin	101	Esculin
35	Schisandrin B (Sch B)	102	Baicalein
36	Diosmetin	103	Cinchonidine
37	Naringin Dihydrochalcone	104	Fisetin
38	Dihydromyricetin	105	Hesperidin
39	(S) -10 -Hydroxycamptothecin	106	Limonin
40	Triptolide (PG490)	107	Nalidixic acid
41	D -Mannitol	108	Osthole
42	Polydatin	109	Quercetin Dihydrate

Table S1 Natural product library

Number	Compound	Number	Compound
43	Sodium Danshensu	110	Silymarin
44	Apocynin	111	Troxerutin
45	Calcium D-Panthotenate	112	Laetrile
46	Cyclocytidine HCl	113	Baicalin
47	Apigenin	114	Cryptotanshinone
48	Berberine HCl	115	Oleuropein
49	Cytisine	116	Palmatine chloride
50	Glycyrrhizin (Glycyrrhizic Acid)	117	Ipriflavone (Osteofix)
51	Icariin	118	Naringin
52	(+) -Matrine	119	Oxymatrine
53	Neohesperidin	120	Rutaecarpine
54	(-)-Parthenolide	121	Sinomenine
55	Salicin	122	Ursolic Acid
56	Tangeretin	123	Andrographolide
57	(-)-Epigallocatechin Gallate	124	Bergenin
58	Arbutin	125	Cyclosporin A
59	Bilobalide	126	Ferulic Acid
60	Dihydroartemisinin (DHA)	127	Hyodeoxycholic acid (HDCA)
61	Gossypol	128	Magnolol
62	Indole -3-carbinol	129	Neohesperidin dihydrochalcone (Nhdc)
63	Methyl -Hesperidin	130	Paeonol
64	Nobiletin	131	Rutin
65	Phloretin	132	Synephrine
66	Sclareol	133	Vanillylacetone
67	Tanshinone I		

Table S2 Top 20 upregulated genes in Que-treated WS hMSCs

Rank	Gene	Description	Log_2(fold change)	padj
1	MASP1	mannan binding lectin serine peptidase 1	6.205	0.000
2	NR5A2	nuclear receptor subfamily 5 group A member 2	4.964	0.034
3	PSG2	pregnancy specific beta-1-glycoprotein 2	4.963	0.032
4	HOXC9	homeobox C9	4.554	0.005
5	PSG4	pregnancy specific beta-1-glycoprotein 4	4.536	0.000
6	PSG5	pregnancy specific beta-1-glycoprotein 5	4.536	0.000
7	CLEC2B	C-type lectin domain family 2 member B	4.137	0.002
8	SHISA7	shisa family member 7	3.915	0.000
9	SCIN	scinderin	3.872	0.000
10	STEAP1	STEAP family member 1	3.871	0.001
11	IGFBP3	insulin like growth factor binding protein 3	3.786	0.000
12	PCSK6	proprotein convertase subtilisin/kexin type 6	3.739	0.000
13	SELENBP1	selenium binding protein 1	3.664	0.018
14	PODN	podocan	3.655	0.000
15	PDE7B	phosphodiesterase 7B	3.617	0.000
16	PSG1	pregnancy specific beta-1-glycoprotein 1	3.568	0.000
17	HCLS1	hematopoietic cell-specific Lyn substrate 1	3.417	0.000
18	GALNT15	polypeptide N-acetylgalactosaminyltransferase 15	3.389	0.000
19	GCNT4	glucosaminyl (N-acetyl) transferase 4, core 2	3.382	0.023
20	PSG9	pregnancy specific beta-1-glycoprotein 9	3.365	0.009

Table S3 Top 20 downregulated genes in Que-treated WS hMSCs

Rank	Gene	Description	Log_2(fold change)	padj
1	C3	complement C3	-5.093	0.000
2	CYP1B1	cytochrome P450 family 1 subfamily B member 1	-4.178	0.000
3	CA8	carbonic anhydrase 8	-4.12	0.003
4	SERPINA1	serpin family A member 1	-4.038	0.005
5	PCLO	piccolo presynaptic cytomatrix protein	-3.993	0.035
6	TENM1	teneurin transmembrane protein 1	-3.842	0.000
7	NPIPA5	nuclear pore complex interacting protein family member A5	-3.579	0.013
8	ADGRF5	adhesion G protein-coupled receptor F5	-3.315	0.024
9	CLEC18B	C-type lectin domain family 18 member B	-3.125	0.043
10	EPGN	epithelial mitogen	-3.007	0.007
11	FOXL2NB	FOXL2 neighbor	-2.755	0.002
12	BEGAIN	brain enriched guanylate kinase associated	-2.623	0.001
13	CCDC3	coiled-coil domain containing 3	-2.515	0.000
14	ZFYVE28	zinc finger FYVE-type containing 28	-2.48	0.004
15	RNF112	ring finger protein 112	-2.467	0.023
16	CADM2	cell adhesion molecule 2	-2.441	0.023
17	SPINT2	serine peptidase inhibitor, Kunitz type 2	-2.414	0.000
18	STRA6	stimulated by retinoic acid 6	-2.402	0.000
19	MAGEB17	MAGE family member B17	-2.398	0.011
20	CCNO	cyclin O	-2.388	0.049

Table S4 Quantitative RT-PCR primers

Gene	Forward primer (5’->3’)	Reverse primer (5’->3’)
18S	GTAACCCGTTGAACCCCATT	CCATCCAATCGGTAGTAGCG
Progerin	ACTGCAGCAGCTCGGGG	TCTGGGGGCTCTGGGC
Lamin B1	GAAAAAGACAACTCTCGTCGCA	GTAAGCACTGATTTCCATGTCCA
IL6	ACTCACCTCTTCAGAACGAATTG	CCATCTTTGGAAGGTTCAGGTTG
LAP2β	CCCCTCGGTCCTGACAAAAG	CGCTCTTCGTCACTGGAGAA
Sat2	CATCGAATGGAAATGAAAGGAGTC	ACCATTGGATGATTGCAGTCAA
α-Sat	CTGCACTACCTGAAGAGGAC	GATGGTTCAACACTCTTACA
36B4	CAGCAAGTGGGAAGGTGTAATCC	CCCATTCTATCATCAACGGGTACAA
Telomere	GGTTTTTGAGGGTGAGGGTGAGGG	TCCCGACTATCCCTATCCCTATCCC
	TGAGGGTGAGGGT	TATCCCTATCCCTA
SOD1	GGTGGGCCAAAGGATGAAGAG	CCACAAGCCAAACGACTTCC
SOD2	TTTCAATAAGGAACGGGGACAC	GTGCTCCCACACATCAATCC
GSR	CACGAGTGATCCCAAGCCC	CAATGTAACCTGCACCAACAATG
GSTP1	CCCTACACCGTGGTCTATTTCC	CAGGAGGCTTTGAGTGAGC
